# "Tandem duplication-random loss" is not a real feature of oyster mitochondrial genomes

**DOI:** 10.1186/1471-2164-10-84

**Published:** 2009-02-19

**Authors:** Jianfeng Ren, Xiao Liu, Guofan Zhang, Bin Liu, Ximing Guo

**Affiliations:** 1 Institute of Oceanology, Chinese Academy of Sciences, Qingdao 266071, PR China; 2 Beijing Institute of Genomics, Chinese Academy of Sciences, Beijing 101300, PR China; 3 The Graduate University of Chinese Academy of Sciences, Beijing 100039, PR China; 4 Haskin Shellfish Research Laboratory, Institute of Marine and Coastal Sciences, Rutgers University, Port Norris, NJ 08349, USA

## Abstract

Duplications and rearrangements of coding genes are major themes in the evolution of mitochondrial genomes, bearing important consequences in the function of mitochondria and the fitness of organisms. Yu *et al*. (*BMC Genomics *2008, 9:477) reported the complete mt genome sequence of the oyster *Crassostrea hongkongensis *(16,475 bp) and found that a DNA segment containing four tRNA genes (*trnK_1_*, *trnC*, *trnQ_1 _*and *trnN*), a duplicated (*rrnS*) and a split rRNA gene (*rrnL5*') was absent compared with that of two other *Crassostrea *species. It was suggested that the absence was a novel case of "tandem duplication-random loss" with evolutionary significance. We independently sequenced the complete mt genome of three *C. hongkongensis *individuals, all of which were 18,622 bp and contained the segment that was missing in Yu *et al*.'s sequence. Further, we designed primers, verified sequences and demonstrated that the sequence loss in Yu *et al*.'s study was an artifact caused by placing primers in a duplicated region. The duplication and split of ribosomal RNA genes are unique for *Crassostrea *oysters and not lost in *C. hongkongensis*. Our study highlights the need for caution when amplifying and sequencing through duplicated regions of the genome.

## Background

Because of its nature of maternal inheritance, mitochondrial (mt) genome has a fast rate of evolution and is particularly useful in phylogenetic analysis. The analysis of complete mt genome sequences provides not only information about nucleotide changes, but also insights into gene order and rearrangements that are indicative of major evolutionary changes.

We read with great interest an article appeared in a recent issue of *BMC Genomics *(9:477 2008) entitled 'Complete mitochondrial DNA sequence of oyster *Crassostrea hongkongensis *– a case of "Tandem duplication-random loss" for genome rearrangement in *Crassostrea*?' by Yu, Z.N., Wei, Z.P., Kong, X.Y., and Shi, W. [1]. Based on our data, we believe that an important part of Yu *et al*.'s paper is incorrect and would like to share our results with the readers of this Journal.

In their paper, Yu *et al*. (2008) reported that the complete mt genome of *C. hongkongensis *is 16,475 bp in length (GenBank accession number EU266073) and pointed out that 'A striking finding of this study is that a DNA segment containing four tRNA genes (*trnK_1_*, *trnC*, *trnQ_1 _*and *trnN*) and two duplicated or split rRNA genes (*rrnL5*' and *rrnS*) are absent from the genome, when compared with that of two other extant *Crassostrea *species, which is very likely a consequence of loss of a single genomic region present in ancestor of *C. hongkongensis*. It indicates this region seem to be a "hot spot" of genomic rearrangements over the *Crassostrea *mt-genomes' (p. 1, Abstract, line 14–19). We have independently sequenced the complete mt genomes of three *C. hongkongensis *individuals. All our three sequences contained the DNA segment that was reported missing in Yu *et al*.'s study. The discrepancy is not trivial as the loss of the duplicated region was central to Yu *et al*.'s hypothesis of a novel "tandem duplication and random loss" event during the evolution of *C. hongkongensis*. It was further suggested that this region was a "hot spot" for genomic rearrangement. Therefore, it is critical to determine if the loss of the duplicated region is real in view of the different sequences we obtained.

To determine which sequence is correct, we analyzed our sequences, compared them with Yu *et al*.'s sequence and sequences from other *Crassostrea *species, and designed primers to test the presence or absence of the genome region in question. Here we report that Yu *et al*.'s sequence is either incorrect or represent a rare mutation that is uncommon in *C. hongkongensis*.

## Experimental design, results and discussion

The three complete mt genome sequences of *C. hongkongensis *that we independently obtained were submitted to GenBank: accession No. EU672834 for oyster HN from Hainan, FJ593172 for oyster BH45 from Guangxi, and FJ593173 for oyster H50 from Fujian. The length of the complete mt genome of *C. hongkongensis *reported by Yu *et al*. is 16,475 bp. The length of all three mt sequences that we obtained is 18,622 bp, which is 2,147 bp longer than that of Yu *et al*.'s. We aligned our sequences with that of Yu *et al*. and other *Crassostrea *species. We annotated our mt sequences according to that of *C. gigas *with minor revisions [2], and the results are presented in Table 1. Our sequence for *C. hongkongensis *has exactly the same gene order and arrangements as *C. gigas*, both containing the segment that is missing in Yu *et al*.'s sequence. The segment contains four tRNA genes, a duplicated *rrnS *and part of the split *rrnL*. The split *rrnL *is first discovered in *C. virginica *and appears to be unique for oysters [2].

**Table 1:** Annotation of the mitochondrial genome of *Crassostrea hongkongensis*.

Gene	Position	Size		Codon		Intergenic Nucleotides*
				
		Nucleotide	Amino acids	Start	Stop	
***cox1***	1–1617	1617	538	ATA	TAA	1
***rrnL ***3'half	1761–2472	712				143
***cox3***	2575–3438	864	287	ATA	TAA	231
***trnI***	3439–3505	67				0
***trnT***	3506–3573	68				0
***trnE***	3595–3662	68				21
***cob***	3670–4875	1206	401	ATA	TAA	130
***trnD***	4984–5052	69				108
***cox2***	5054–5755	702	233	ATG	TAG	1
***trnM*_*1*_^(*ATG*)^**	5777–5842	66				21
***trnS*_*1*_^(*AGN*)^**	5846–5915	70				3
***trnL*_*2*_^(*UUR*)^**	5931–5997	67				15
***trnM*_*2*_^(*ATG*)^**	6065–6129	65				67
***trnS*_*2*_^(*UCN*)^**	6137–6204	68				7
***trnP***	6383–6451	69				178
***rrnS***_*1*_	6452–7525	1074				0
***trnK***_*1*_	7526–7594	69				0
***trnC***	7624–7691	68				29
***trnQ*_*1*_^(*CAA*)^**	7709–7777	69				17
***rrnL ***5'half	7780–8384	605				1
***trnN***	8443–8508	66				58
***rrnS***_*2*_	8509–9698	1190				0
***trnY***	9699–9764	66				0
***atp6***	9770–10453	684	227	ATG	TAG	5
***trnG***	10966–11035	70				512
***trnV***	11644–11716	73				608
***nad2***	11759–12757	999	332	ATG	TAG	42
***trnR***	12792–12858	67				34
***trnH***	12919–12983	65				60
***nad4***	12986–14335	1350	449	ATA	TAG	2
***trnK*_*2*_^(*AAA*)^**	14343–14417	75				7
***nad5***	14419–16089	1671	556	ATG	TAA	1
***nad6***	16101–16576	476	158	ATT	TA-	11
***trnQ*_*2*_^(*CAA*)^**	16610–16678	69				33
***nad3***	16684–17034	351	116	ATG	TAG	5
***trnL*_*1*_^(*CUN*)^**	17070–17135	66				35
***trnF***	17171–17238	68				35
***trnA***	17259–17325	67				20
***nad1***	17331–18266	936	311	ATG	TAA	5
***nad4L***	18270–18549	280	93	ATG	T-	3
***trnW***	18550–18618	69	22			0

"-" indicates termination codons completed via polyadenylation.

Underlined genes correspond to a segment missing in Yu *et al*.' sequence due to a sequencing artifact.

The three *C. honghongensis *oysters used in our study were from diverse populations (Hainan, Guangxi and Fujian) covering the entire geographic range of this species as we know (Guo *et al*., unpublished), and they were genetically identified using molecular markers prior to our study [3]. We compared one of our sequences with Yu *et al*.'s using BLAST [4]. In the 16,475 shared nucleotides, there are 15 SNPs (single nucleotide polymorphisms) and the similarity between the two gnomes is 99.91%, suggesting that oysters used in our study and Yu *et al*.'s study are all *C. hongkongensis*. Sequence identity in major coding genes between our *C. hongkongensis *sequences and that of C. gigas is shown in Table 2. Considerable differentiation has occurred between the two sister-species at some genes (i.e., gene identity of 75.1% for nad2) despite the identical gene order. Analysis of all four *C. hongkongensis *mt sequences revealed 41 SNPs: 28 in coding and 13 in non-coding regions (Table 3). Of the 19 SNPs from protein coding genes, only one is non-synonymous, suggesting strong purifying selection. The non-synonymous mutation occurred at the *atp6 *gene in Yu's sequence only, and further studies are needed to determine whether it is a true SNP or sequencing error.

**Table 2:** Sequence identity of major coding genes between *Crassostrea hongkongensis *and *C. gigas*.

Gene	Number of nucleotides		Identity (%)	Number of amino acids		Identity (%)
				
	*C. hongkongensis*	*C. gigas*		*C. hongkongensis*	*C. gigas*	
*cox1*	1617	1617	87.4	538	538	98.0
*cox2*	702	702	87.6	233	233	98.7
*cox3*	864	876	82.7	287	291	89.7
*nad1*	936	936	82.7	311	311	87.8
*nad2*	999	999	75.1	332	332	72.6
*nad3*	351	351	82.3	116	116	86.2
*nad4*	1350	1353	78.3	449	450	81.8
*nd4L*	280	283	82.3	93	94	93.5
*nad5*	1671	1671	77.1	556	556	80.2
*nad6*	476	477	77.6	158	158	77.2
*cob*	1223	1248	79.5	401	412	88.0
*atp6*	684	684	84.5	227	227	97.4
*rrnS*_*1*_	1074	1037	93.2			
*rrnS*_*2*_	1190	1205	89.9			
*rrnL *5'	605	601	88.4			
*rrnL *3'	712	713	95.8			

**Table 3:** Single-nucleotide polymorphism (SNP) observed among four mitochondrial genome sequences of *Crassostrea hongkongensis*.

**Gene**	**Position**	**HN**	**BH45**	**HC50**	**Yu^1^**	**Type**
*cox1 *(1–1617)	930	C	C	A	C	Transversion, synonymous
	993	C	C	C	T	Transition, synonymous
	1395	T	T	T	C	Transition, synonymous
*cox3 *(2575–3438)	2805	G	A	G	G	Transition, synonymous
	3282	G	T	G	G	Transversion, synonymous
*cob *(3670–4875)	4611	T	C	T	T	Transition, synonymous
*cob-trnD *(4876–4983)	4883	C	C	C	T	Transition
*cox2 *(5054–5755)	5533	A	G	G	G	Transition, synonymous
*cox2-trnS*_*1*_(5756–5845)	5845	T	G	G	G	Transversion
*rrnS*_*1*_(6452–7525)	7225	C	T	C	-	Transition
	7325	C	C	T	-	Transition
*rrnL-trnN *(8385–8442)	8407	T	C	T	-	Transition
*rrnS*_*2 *_(8509–9698)	8567	C	T	C	-	Transition
	8832	A	A	A	G	Transition
	9036	G	G	T	G	Transversion
	9219	T	C	T	T	Transition
	9372	C	T	C	C	Transition
*atp6 *(9770–10453)	10025	T	T	T	C	Transition, nonsynonymous
*atp6-trnG *(10454–10965)	10524	G	T	G	G	Transversion
	10673	G	A	G	G	Transition
	10712	C	C	T	C	Transition
	10778	A	G	G	G	Transition
	10836	C	T	C	C	Transition
	10870	T	C	T	T	Transition
*trnG-trnV *(11036–11643)	11502	C	G	G	G	Transversion
	11509	C	T	T	T	Transition
	11638	T	C	T	T	Transition
*trnV *(11644–11716)	11699	T	C	T	C	Transition
	11701	G	A	A	A	Transition
*nad2 *(11759–12757)	12070	A	G	A	A	Transition, synonymous
*nad4 *(12986–14335)	13232	T	C	T	T	Transition, synonymous
	13678	C	A	C	C	Transversion, synonymous
*nad5 *(14419–16089)	15159	A	G	G	G	Transition, synonymous
	15216	T	C	T	T	Transition, synonymous
	15321	G	G	T	G	Transversion, synonymous
	15552	A	A	A	G	Transition, synonymous
	15645	T	T	C	T	Transition, synonymous
*nad6-trnQ2 *(16577–16609)	16590	C	C	T	C	Transition
*nad3 *(16684–17034)	16986	A	G	G	G	Transition, synonymous
*nad1 *(17331–18266)	17876	T	C	T	T	Transition, synonymous
	18134	A	A	G	A	Transition, synonymous

^1^Sequence for Yu is from Yu et al., 2008, and the other three sequences are from this study.

**Figure 1:**
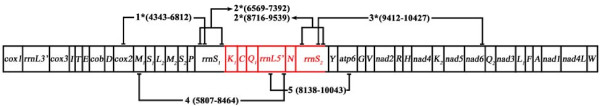
**Position map of the primers used in amplifying fragments of *C. hongkongensis *mitochondrial genome**. Above the gene map are the three pairs of primers used by Yu *et al*. (2008) and below are the two pairs of primers designed to confirm the existence of the gene block in red, which is reported missing by Yu *et al*. Gene segments are not drawn to scale.

Yu *et al*. used ten pairs of primers to amplify the complete mt genome of *C. hongkongensis *(p. 11). We carefully studied the positions of each primer and located them in our mt genome sequences of *C. hongkongensis *(Figure 1). It occurred to us that Yu *et al*. might have failed to amplify the gene block of *K_1_-C-Q_1_-rrnL*5'-*N*-*rrnS*_*2 *_because some of their primers were placed in a duplicated region. As shown in Figure 1, primer pair 1* is located in gene *cob *and *rrnS*_*1 *_(or *rrnS*_*2*_), primer pair 2* is completely located within the duplicated gene *rrnS *(*rrnS*_*1 *_or/and *rrnS*_*2*_), and primer pair 3* is located in *rrnS*_*2 *_(or *rrnS*_*1*_) and *atp6 *(primer pairs 1*, 2* and 3* correspond to the third, the fourth and the fifth primer pairs in Yu *et al*.' paper, Table 4). Because these three primer pairs are either completely or partially (one of the two primers) located in the duplicated gene *rrnS*_*1 *_and *rrnS*_*2*_, they should theoretically amplify two fragments of different length, but in reality the smaller fragment may be preferentially amplified and sequenced. The length of shortest PCR products expected from the three primer pairs was 2,470 bp, 824 bp and 1,016 bp, respectively (Table 4). Primer pair 2* was completely located in the duplicated gene *rrnS *(*rrnS*_*1 *_or *rrnS*_*2*_); thus they may directly concatenate the sequence between the duplicated gene and artificially lose the gene block of *K1-C-Q1-rrnL*5'-*N*-*rrnS*_*2 *_(Figure 1). The block, 2,147 bp, may be too large to be amplified under competition with a smaller fragment.

**Table 4:** Primers used to amplify fragments of *Crassostrea hongkongensis *mitochondrial genome.

Order	Primer name	Sequence (5'-3')	Length	position	Product size (bp)
1*	HK-4343F	TTAGAGTTCCGTTTCACCCG	20	4343	2470, 4617
	HK-6812R	CTTTCGCTTCAATTTAGTTAGT	22	6812, 8959	
2*	HK-6569F	GGTTCTGGTATAATGTTAGCT	21	6569, 8716	824, 2971
	HK-7392R	ATTACTCTCTTTTTACTCCC	20	7392, 9539	
3*	HK-9412F	CTAGGTCAGGTCGAAGTGCT	20	7265, 9412	1016, 3163
	HK-10427R	AGAGCACAGGTGTTGGGTGA	20	10427	
4	HK-5807F	GTCTCATAATCCGAAAGTGGTT	22	5807	2658
	HK-8464R	CTTATACTTGGGCTACTTTCTT	22	8464	
5	HK-8138F	GGTGCTCACTAAATCAGTATGT	22	8138	1905
	HK-10043R	ATGAAGATAGTGACGGAAACCC	22	10043	

*Primer pairs from Yu *et al*. (2008). Because one or both of the primers are located in the duplicated *rrnS *gene, two fragments are expected but only the shorter one is actually amplified.

**Figure 2:**
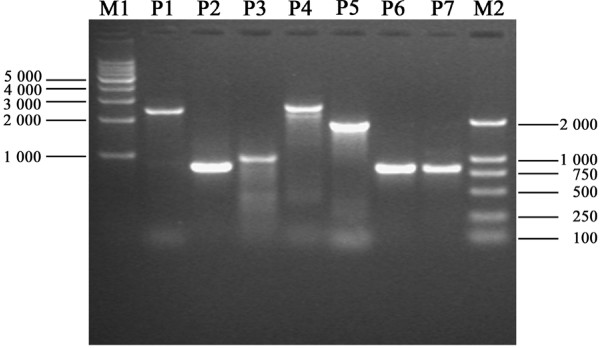
**PCR products amplified with different primers and separated on agarose gel electrophoresis**. P1 - P3 are the products amplified using the primer pairs 1* – 3* and P4, P5 are the products amplified with the primers 4, 5 with genomic DNA template; while P6, P7 are the products amplified using the primers 2* with P4 and P5 as templates, respectively. M1: 1 Kb DNA Ladder marker 10000, 8000, 7000, 6000, 5000, 4000, 3000, 2000 and 1000 bp; M2: D2000 marker 2000, 1 000, 750, 500, 250 and 100 bp.

To test our hypothesis that the gene block between duplicated *rrnS *failed to amplify in Yu *et al*.'s study, we synthesized the three primer pairs used by Yu *et al*. (after removing mismatches based on our *C. hongkongensis *sequences to improve specificity). As expected, the three shorter products mentioned in Yu *et al*.' paper were successfully obtained (Table 4, Figure 2). We increased the elongation time for PCR trying to obtain the longer fragments, but failed probably because of distance between the duplicated genes (2,147 bp) is too long. We designed two new pairs of primers targeting the block between the duplicated *rrnS *genes, with one primer of each pair located in the *rrnL *gene that was supposed to be absent according to Yu *et al*. (Table 4, Fig, 1). The two new primer pairs designed by us successfully amplified and produced fragments of expected sizes, 2,658 and 1,905 bp (Table 4, Figure 2), proving that the gene block between the duplicated *rrnS *genes are actually there. To further confirm that the two products both contain the duplicated *rrnS*, each product was used as PCR template for amplification with the primers 2* that amplifies *rrnS *only; both PCR produced a fragment of the expected size (824 bp), the same as using genomic DNA as template (Figure 2). We also sequenced some of the fragments, and the sequences are the same as expected from the mt sequences we obtained. These results clearly demonstrate that the duplicated *rrnS *and the split *rrnL *exist in the mt genome of *C. hongkongensis*. There is no loss of the duplicated genes and the gene block between them. "Tandem duplication-random loss" is not a real feature of oyster mt genomes and has not occurred during the evolution of *C. hongkongensis*. The possibility of Yu *et al*. sequenced a rare mutant of *C. hongkongensis *is extremely low considering: 1) we sequenced three individuals from three diverse populations; 2) Yu and colleagues screened more than one individual; and 3) we duplicated their results with our samples. This is a clear case of PCR artifacts involving duplicated genes.

## Conclusion

In conclusion, the complete mt genome of *C. hongkongensis *is 18,622 bp in length, and its gene order and arrangement are identical to that of *C. gigas*. The loss of a gene segment reported by Yu *et al*. (2008) was an artifact due to placing PCR primers in a duplicated gene, and the phenomenon of "tandem duplication-random loss" does not exist in the mt genome of *C. hongkongensis*. Our study highlights the need for caution when amplifying and sequencing through regions with tandem duplication. When tandem duplication is expected, it is important to design long PCR fragments and not place primers in duplicated regions. Cross-verification with different sets of primers should be considered.

## Authors' contributions

JR did PCR, sequencing and initial analysis; XG and XL provided the samples; BL, XG, LX and GZ conceived the study; JR, BL and XG did most of the writing. All authors read and approved the final manuscript.
